# Is there a role for expectation maximization imputation in addressing missing data in research using WOMAC questionnaire? Comparison to the standard mean approach and a tutorial

**DOI:** 10.1186/1471-2474-12-109

**Published:** 2011-05-23

**Authors:** Hassan MK Ghomrawi, Lisa A Mandl, John Rutledge, Michael M Alexiades, Madhu Mazumdar

**Affiliations:** 1 Department of Public Health, Weill Cornell Medical College, New York, USA; 2 Hospital For Special Surgery, New York, USA; 3 Center for Cancer Research, The Valley Hospital, New Jersey, USA

## Abstract

**Background:**

Standard mean imputation for missing values in the Western Ontario and Mc Master (WOMAC) Osteoarthritis Index limits the use of collected data and may lead to bias. Probability model-based imputation methods overcome such limitations but were never before applied to the WOMAC. In this study, we compare imputation results for the Expectation Maximization method (EM) and the mean imputation method for WOMAC in a cohort of total hip replacement patients.

**Methods:**

WOMAC data on a consecutive cohort of 2062 patients scheduled for surgery were analyzed. Rates of missing values in each of the WOMAC items from this large cohort were used to create missing patterns in the subset of patients with complete data. EM and the WOMAC's method of imputation are then applied to fill the missing values. Summary score statistics for both methods are then described through box-plot and contrasted with the complete case (CC) analysis and the true score (TS). This process is repeated using a smaller sample size of 200 randomly drawn patients with higher missing rate (5 times the rates of missing values observed in the 2062 patients capped at 45%).

**Results:**

Rate of missing values per item ranged from 2.9% to 14.5% and 1339 patients had complete data. Probability model-based EM imputed a score for all subjects while WOMAC's imputation method did not. Mean subscale scores were very similar for both imputation methods and were similar to the true score; however, the EM method results were more consistent with the TS after simulation. This difference became more pronounced as the number of items in a subscale increased and the sample size decreased.

**Conclusions:**

The EM method provides a better alternative to the WOMAC imputation method. The EM method is more accurate and imputes data to create a complete data set. These features are very valuable for patient-reported outcomes research in which resources are limited and the WOMAC score is used in a multivariate analysis.

## Background

Since its development by Bellamy and colleagues, the Western Ontario and Mc Master (WOMAC) Osteoarthritis Index has been widely used in assessing functional disability of the hip and the knee [1,2]. The WOMAC has 24 items that represent 3 subscales (function 17 questions, stiffness 2 questions and pain 5 questions). Over the years, its validity has been established for a large number of lower extremity conditions. In fact, it has become one of the standard survey instruments to assess joint-specific function, pain and stiffness in orthopedic outcome studies [3].

As a survey instrument, the WOMAC is susceptible to the problem of missing data since not every patient filling out the WOMAC is likely to provide answers for all the survey items. Bellamy and colleagues have recognized this problem early on and developed an imputation algorithm that addresses missing values. Their method is a variant of the standard mean imputation method, which suggests that the user should substitute the average value for the subscale in lieu of the missing item value(s) whenever items are missing up to a certain maximum; otherwise a subscale score will not be calculated. Because the method of Bellamy et al. (hereafter the WOMAC method) is relatively simple and very intuitive, it is widely used among orthopedic outcomes researchers.

Despite its appeal, the WOMAC method can be limiting for a number of reasons. If the rate of missing values exceeds the predefined maximum, the available data are considered of no value and omitted from any analysis. This limitation has the potential to produce biased estimates of variance and covariance when certain assumptions regarding randomness or normal distribution of missing entries are violated. This may result in a biased perspective of the severity of osteoarthritis in the studied population [4]. Moreover, in cases where the WOMAC is used alongside other survey instruments that may suffer similar problems in regards to missing values (i.e. Short-Form 36), data for fewer subjects than originally anticipated will be used in the analysis [5]. A direct consequence of this loss of data is a reduction in sample size and the need to recruit additional subjects with unplanned additional effort and cost. An alternative to the widely used WOMAC questionnaire is the short form WOMAC that has a shorter function subscale (8 vs. 17 questions). Although a smaller number of questions has the potential to reduce the likelihood of missing values, it does not eliminate it [6].

A substantial body of statistical literature has long existed that addresses missing values utilizing probabilistic models [7]. Some of these imputation methods can remedy this problem because they make assumptions about the distribution of the data and impute missing values based on this distribution. With the advances in computational capabilities, many of these methods have become available in standard statistical packages such as SAS, SPSS and R. Surprisingly, little attention has been given to addressing missing values in quality of life research in orthopedics (a Medline search with key word of 'missing data' and 'orthopedics' retrieves only 2 studies as opposed to 51 studies for 'missing data' and 'oncology'). We are aware of no studies which examined the added value of using advanced probability-based imputation methods as compared to the WOMAC method.

The main purpose of this paper was to investigate the performance of one probabilistic imputation method, the expectation maximization (EM) method, as compared to the WOMAC method using data from a large cohort of total hip replacement (THR) patients. A secondary purpose of this paper was to provide a tutorial on using EM for the WOMAC in SPSS.

## Methods

Our study sample consisted of patients who, after providing informed consent, participated in a prospective THR registry at the Hospital for Special Surgery (HSS), New York, and accrued between April 30th, 2007 and October 3rd, 2008. The registry is approved by the HSS Institutional Review Board and conforms to the Helsinki Declaration. Primary total hip replacement patients filled out the Hip disability and Osteoarthritis Outcome Scores questionnaire (HOOS) [8-10] as part of a battery of preoperative surveys. We calculated the WOMAC subscale scores of pain, stiffness and function from the HOOS questionnaire data. The HOOS includes the WOMAC in its complete form in addition to other questions about more strenuous activities. The 5 'Pain' questions in WOMAC were embedded with 5 additional questions in the HOOS pain subscale; 2 Stiffness questions of WOMAC embedded in 5 'Symptoms' related questions in HOOS; and the 17 'Physical Function' related questions were exactly the same in both questionnaires.

A univariate analysis was conducted on each individual WOMAC item in the attained data set to calculate the item-specific rate of missing values. An analytic data set was then created by deleting all subjects with any incomplete responses; this dataset thus contains only subjects with complete information whereby the true score was known for all subjects.

For each item in the analytic dataset, we introduced missing values at the same rate as observed in the attained dataset by randomly drawing subjects and deleting their data for that item. The goal was to recreate the same missing data percentage item by item as observed in original attained dataset. To compare methods, we first calculated the scores for the 3 subscales for complete cases (CC), i.e. the sum of item scores in each subscale for the subset of the analytic dataset that contains only cases with complete information. The purpose of this step is to set a baseline to which we compared the added value of the 2 imputation methods. Subsequently we employed the WOMAC imputation method, and the EM imputation method.

The WOMAC method is a variant of a standard mean imputation method. In the case of missing data, scores of the non-missing items for each case were added and the mean value was used to impute for the missing values. However, if the patient has not replied to one or two stiffness questions, one or two of the five pain questions or four or more of the 17 physical function questions were considered non-scorable.

The EM imputation method is a deterministic iterative algorithm that determines the maximum likelihood estimates of the parameters of the distribution which the complete (missing and observed) data are assumed to follow. We assumed that the data followed a multivariate normal distribution. At each iteration, in the first step (E-step), the conditional expectation of the log-likelihood of the complete data is evaluated, where the expectation is taken with respect to the distribution of the missing data conditional on the observed data and the parameters estimated at the previous iteration. In the second step (M-step), the expected log-likelihood evaluated in the E-step is maximized and new estimates for the parameters are obtained. The iterations are repeated until convergence is reached.

This exercise of creating missing data, calculating CC, WOMAC method, and EM subscale scores was repeated 1000 times in order to observe the variability and sensitivity of the results. For each time, the 3 subscale scores as well as the number of patients for which a score was successfully computed were recorded. Since the percent missing in our data was low and the cohort size was much larger than a conventional orthopedic study [3], we re-analyzed our data using a sample of 200 randomly selected patients from our analytic dataset. We also created missing values at random that were 5 times the rates observed in our original attained dataset, however, capping the rate of missing values at 45% to account for the items which had high rates of missing values.

The results are displayed in a box-plot, which is a graphical way to depict summary statistics for data: a rectangle is drawn that extends from the first quartile to the third quartile, with a bar that identifies the median value. The height of the rectangle thus indicates the inter-quartile range (IQR). In addition, two whiskers are drawn: the upper whisker corresponds to the largest observed value within 1.5 times the IQR above the third quartile, and the lower whisker corresponds to the smallest observed value within 1.5 times the IQR below the first quartile. Observed values outside the two whiskers (outliers) are drawn as points.

## Results

Two thousand and sixty two (2062) THR patients were enrolled in the registry between April 30, 2007 and October 3, 2008. Half (50%) of all THR patients were female with a mean age of 62 years (range: 18 - 102) and 92% were white. Table 1 displays the mean and standard deviation of scores and prevalence of missing values in each of the WOMAC items in the attained data set. Rate of missing values was generally low (<5%) for most of the items. However, few items had higher prevalence of missing values including descending stairs, standing, getting in and out of bath, being involved in heavy domestic duties (moving heavy boxes, scrubbing floors etc.). Consequently, 1339 (64.9%) patients of the 2062 patients had completed all items of the WOMAC. Average subscale scores were 9.3 for the pain scale, 4.2 for the stiffness subscale, and 33.4 for the function subscale.

**Table 1:** Mean scores and percent missing for the individual WOMAC items

	Entire Cohort (N = 2062)	
	**Mean Score (SD)**	**% Missing**

**Pain Questions**:		

Walking on a flat surface	2.0 (0.9)	3.9

Going up or down stairs	2.3 (0.9)	4.4

At night while in bed	1.8 (1.0)	3.5

Sitting or lying	1.5 (1.0)	3.9

While Standing	1.7 (0.9)	3.8

**Stiffness Questions**:		

How severe is your knee joint stiffness after first wakening in the morning?	2.1 (1.0)	3.4

How severe is your knee stiffness after sitting, lying or resting later in the day?	2.1 (0.9)	2.9

**Difficulty in Function Questions**:		

Descending stairs	1.8 (1.0)	4.7

Ascending stairs	2.1 (1.0)	4.1

Rising from sitting	2.0 (0.9)	3.2

Standing	1.7 (0.9)	4.0

Bending to the floor/pick up an object	2.2 (1.0)	3.7

Walking on a flat surface	1.8 (0.9)	3.8

Getting in/out of car	2.3 (0.9)	3.7

Going shopping	2.0 (0.9)	6.5

Putting on socks/stockings	2.4 (1.0)	4.1

Rising from bed	1.9 (1.0)	3.4

Taking off socks/stockings	2.2 (1.0)	4.6

Lying in bed (turning over, maintaining hip position)	2.0 (1.0)	3.3

Getting in/out of bath	1.7 (1.0)	12.3

Sitting	1.4 (0.9)	3.8

Getting on/off toilet	1.7 (1.0)	3.3

Heavy domestic duties (moving heavy boxes, scrubbing floors etc.)	2.6 (1.0)	14.5

Light domestic duties (cooking, dusting etc.)	1.6 (0.9)	7.1

**Figure 1:**
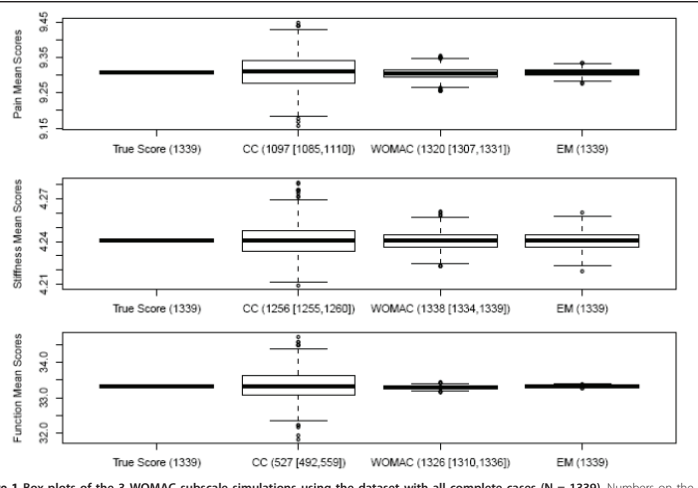
**Box-plots of the 3 WOMAC subscale simulations using the dataset with all complete cases (N = 1339)**. Numbers on the X-axis refer to the median and range of samples considered in the score evaluation for different imputation methods. Numbers on the Y-axis represent the range of scores of a particular subscale.

The imputation results for the different methods applied to the analytic dataset (N = 1339) are displayed as box-plots in Figure 1. In all three subscales, the box-plot for the complete cases showed a significant loss of subjects for which a subscale score was attainable (1097/1339 for pain, 1256/1339 for stiffness, and 527/1339 for function) as well as a relatively wide variability in the attained scores. The range of this variability narrowed noticeably upon imputation using either imputation method. In addition, scores were also attainable for the large majority of patients using the WOMAC method (1307/1339 for pain, 1338/1339 for stiffness, and 1326/1339 for function) and for all the patients using the EM method.

**Figure 2:**
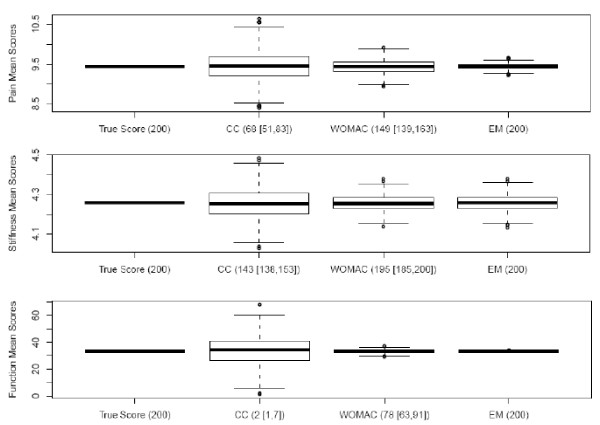
**Box-plots of the 3 WOMAC subscale simulations using the dataset with a random sample of complete cases (N = 200) and 5 times the missing rate**. Numbers on the X-axis refer to the median and range of samples considered in the score evaluation for different imputation methods Numbers on the Y-axis represent the range of scores of a particular subscale.

Figure 2 displays the results of the random sample of 200 patients with 5 times the rate of missing values. The same pattern of narrowing ranges was observed both, for the WOMAC and the EM method compared to complete cases. However, in this sample, i.e. as the level of missing values increased, we observed that the WOMAC imputation was able to calculate the subscale scores for a smaller number of subjects in this subset (N = 200) compared to those in the analytic dataset (N = 1339) with the exception of the stiffness subscale (pain calculated for 149 of 200 subjects, stiffness calculated for 195 of 200 subjects, and function calculated for 78 of 200 subjects). On the other hand, EM method calculated the subscale scores for all patients.

In both Figure 1 and Figure 2, one can clearly notice a common pattern of reduced range of variability in the scores for the WOMAC method and the EM method as the number of items increased from 2 for stiffness, to 5 for pain to 17 for function, which was not observed for the WOMAC method.

## Discussion

In a climate that is geared towards emphasizing patient-reported outcomes and proper methods for comparative effectiveness research in the field of musculoskeletal disorder, accurate assessment of patients' function will be crucial for evaluating the impact of standard and novel clinical and surgical interventions. The prevalence of missing values in the WOMAC instrument was relatively high with only 64.9% of the patients having complete data, thus a validated imputation method to address missing values in this field is deemed necessary.

This paper evaluated the added value of the EM imputation method (which is based on probabilistic models) as compared to the WOMAC method. Both methods proved effective in imputing for missing values in the large analytic dataset (n = 1339). Yet, our results have shown 2 important advantages of the EM method over WOMAC imputation method when the rate of missing values increases. First, the EM method imputed all missing data for all sampled subjects irrespective of the number of missing values and sample size and demonstrated the ability to calculate subscale scores for all subjects with a small range of variability. This was in contrast to the WOMAC imputation method, whose ability to impute was dependent on the rate of missing values.

To our best knowledge, this is the first study to evaluate the value of an imputation method alternative to the WOMAC method. In this patient population, the EM proved superior to the currently used WOMAC method on several aspects in the analytic dataset. We also simulated a more realistic scenario by employing a smaller sample size and a higher missing rate. However, unlike conventional imputation practices which seek to estimate parameters with missing data that are comparable to population parameters, we focused on recovering lost sample variance and not lost population variance. Indeed, the sample may not be representative of the TKA population. The rate of missing values observed in this cohort of patients was considerably lower than what is usually observed in other studies [11-13], despite the large number of patients and the use of an extensive multi- instrument baseline survey. This lack of representativeness of our sample, although such sample were large and representative of a hospital's population, is a limitation that needs to be addressed in further population-level surveys.

Another limitation of the approach is the assumption of normality of the data. Likert scale items are rarely normally distributed with patient samples. While Schafer's CAT approach, which uses item values as discrete, can be more suitable for categorical values, it is computationally more demanding and is less available in commercial software. We have applied the CAT approach to a subsample of the data and found very similar results between both methods. Additionally, we attempted employing the EM algorithm for imputation with multinomial distribution which is the accepted underlying form for the Likert scale data. However, the algorithm, which is computationally more difficult, took more than a day to run on a regular computer and did not result in much different outcome. While it may be a more realistic in the context of quality of life measures, it will be hard to use it routinely in practice. Aiming to provide a user-friendly solution to the practitioner using the WOMAC instrument, we believe that the benefit in estimating an EM algorithm with multivariate normal distribution is advantageous to the mean imputation method and outweighs the impediments of estimating a more computationally demanding model that may provide only a marginal benefit.

Finally, missing values were created at random in all our analyses, an assumption which complies with the requirements for applying EM. It is likely that some of the observed missing values were not missing at random, and thus our simulation may not have been accurate in reflecting the real world experience. The WOMAC imputation method may be similarly inaccurate because it ignores that non-random nature of missing values. The WOMAC imputation method only addresses the number of missing values within a subject, not patterns across subjects. We are aware of other imputation methods, such as the multiple imputation method, that overcome the non-random missing patterns. However, our choice of the EM method is based on its relative ease of use in already available packages, the fact that EM relies on fewer assumptions compared to the multiple imputation method, and because it may be easier to interpret by practitioners.

## Conclusions

In conclusion, the EM method of imputation effectively maximized the use of collected data, thus overcoming one of the most commonly faced problems in clinical patient survey research. This finding may have important implications in regard to survey collection procedures and resource allocation. More importantly, our results may have considerable consequences in multivariate regression analysis using the WOMAC score as a predictor in terms of increased power and goodness-of-fit. Of equal importance is the finding that the EM method provided a more consistent estimate with a narrower confidence interval compared to the WOMAC method. Precision increased as the number of items in a particular subscale increased. This important characteristic of EM imputation may contribute to a more precise estimate of the effect of various treatments on function. With EM now included as a standard function in widely used statistical packages such as SPSS and SAS, researchers using the WOMAC have the opportunity to maximize the use of their data by using the EM method [14,15], Although we apply this new method to WOMAC other patient surveys will also benefit from the application of this method to address missing values.

### EM Imputation for the WOMAC in SPSS: A Tutorial

Data should be first imported into SPSS. From the **ANALYZE **menu, one should select **MISSING VALUE ANALYSIS**. A window will appear with prompts to enter variables into either the **CATEGORICAL VARIABLES **space or the **QUANTITATIVE VARIABLES **space. WOMAC items for each scale separately should be entered into the **QUANTITATIVE VARIABLES **list and **EM **selected from the **ESTIMATION **options on the right-hand side. The user then clicks on the **EM **tab below the **ESTIMATION **options. A new window will open that allows specifying the **DISTRIBUTION. NORMAL DISTRIBUTION **should be selected. The researcher is strongly advised to take advantage of the **SAVE **option, available on this window, to save the imputed data into a separate file to avoid any permanent imputations to the original data. Once all the options have been selected, the researcher may either execute the commands directly or choose to save the syntax by clicking the **PASTE **option. We have provided below the SPSS syntax for EM imputation:

 MVA

 VARIABLES = ***LIST OF VARIABLE NAMES***

 /EM ( TOLERANCE = 0.001 CONVERGENCE = 0.0001 ITERATIONS = 40

OUTFILE='***FILE NAME***' ).

Where,

***LIST OF VARIABLE NAMES ***includes names of all WOMAC items for the 3 domains

***FILE NAME ***is the full name (including location) of the file which has the data

***ITERATIONS ***is the maximum iterations that the program runs before it terminates. We have set it to 40 since the EM algorithm failed to converge with a lower maximum number of iterations.

### Missing Data Patterns

To determine whether the EM assumption is missing at random or not, the researcher should examine the SPSS output. To establish this possibility, the researcher needs to proceed to the table labeled Separate Variance t Tests. If all the p values exceed .05 or alpha, the data are missing at random [16].

## Competing interests

The authors declare that they have no competing interests.

## Authors' contributions

HG, JR and MM performed most of the data analysis and manuscript preparation. LM and MA participated in conceiving the study design and providing subject matter expertise. All authors read and approved the final manuscript.

## Pre-publication history

The pre-publication history for this paper can be accessed here:

http://www.biomedcentral.com/1471-2474/12/109/prepub
